# Anti-Inflammatory Effects of Bioactive Compounds from Seaweeds, Bryozoans, Jellyfish, Shellfish and Peanut Worms

**DOI:** 10.3390/md21100524

**Published:** 2023-09-30

**Authors:** Md Khursheed, Hardik Ghelani, Reem K. Jan, Thomas E. Adrian

**Affiliations:** College of Medicine, Mohammed Bin Rashid University of Medicine, and Health Sciences, Dubai P.O. Box 505055, United Arab Emirates; md.khursheed@mbru.ac.ae (M.K.); hardik.ghelani@mbru.ac.ae (H.G.); reem.jan@mbru.ac.ae (R.K.J.)

**Keywords:** anti-inflammatory activity, inflammatory pathways, marine drugs, macroalgae, marine seaweeds, bryozoans, medusozoa, mollusks

## Abstract

Inflammation is a defense mechanism of the body in response to harmful stimuli such as pathogens, damaged cells, toxic compounds or radiation. However, chronic inflammation plays an important role in the pathogenesis of a variety of diseases. Multiple anti-inflammatory drugs are currently available for the treatment of inflammation, but all exhibit less efficacy. This drives the search for new anti-inflammatory compounds focusing on natural resources. Marine organisms produce a broad spectrum of bioactive compounds with anti-inflammatory activities. Several are considered as lead compounds for development into drugs. Anti-inflammatory compounds have been extracted from algae, corals, seaweeds and other marine organisms. We previously reviewed anti-inflammatory compounds, as well as crude extracts isolated from echinoderms such as sea cucumbers, sea urchins and starfish. In the present review, we evaluate the anti-inflammatory effects of compounds from other marine organisms, including macroalgae (seaweeds), marine angiosperms (seagrasses), medusozoa (jellyfish), bryozoans (moss animals), mollusks (shellfish) and peanut worms. We also present a review of the molecular mechanisms of the anti-inflammatory activity of these compounds. Our objective in this review is to provide an overview of the current state of research on anti-inflammatory compounds from marine sources and the prospects for their translation into novel anti-inflammatory drugs.

## 1. Introduction

The vast diversity of the marine ecosystem ensures the availability of a wide range of bioactive compounds with biological effects in various disease conditions. There is a diverse range of species found in the marine ecosystem. Indeed, 14 out of 35 animal phyla are exclusively found in the marine environment [1]. This variation creates different marine habitats that are hotspots of biodiversity. Moreover, this biodiversity is not constant but is dynamic in nature [2]. Several efforts have been made to make a census of marine biodiversity in different regions of the world so that we can obtain the benefits of region-specific marine biodiversity. These efforts and challenges have previously been reviewed [3].

Marine biodiversity has motivated researchers across the world to investigate novel compounds that may be valuable for the treatment of various disease conditions. Bioactive compounds have been identified and extracted from various marine organisms and shown to be useful in various pathological conditions as described in a recent review [4]. Interestingly, many of these compounds with valuable pharmaceutical properties are in different phases of preclinical and clinical investigation [5]. Bioactive compounds from marine sources have shown immunomodulatory effects [6], activity against diseases such as type 2 diabetes mellitus [7] or anti-cancer properties [8,9]. Many bioactive compounds show anti-inflammatory activity [10,11]. For example, Frondanol is a sea-cucumber-derived intestinal extract that exhibits anti-inflammatory properties in a dextran sodium sulfate (DSS)-induced colitis mouse model [12]. Notably, several marine-derived compounds appear valuable in inflammatory bowel disease (IBD) [13].

Bioactive compounds have distinct chemical and functional properties [14]. In a previous review, we summarized the chemical and anti-inflammatory properties of major bioactive compounds from echinoderms (sea cucumbers, sea urchins, and starfish) [15]. However, there are other major marine species that produce bioactive compounds and few of these are approved for clinical use. The current review summarizes the anti-inflammatory properties of compounds derived from species other than echinoderms.

## 2. Methods

For this proposed review article preparation, we searched the keywords “Seaweed” + “Anti-inflammatory”, “Bryozoans” + “Anti-inflammatory”, “Jellyfish” + “Anti-inflammatory”, “Shellfish” + “Anti-inflammatory” and “Peanut worm” + “Anti-inflammatory” in PubMed, Scopus, Web of Science, American Chemical Society, Elsevier, MDPI and Springer database from 2010 to 2023. The inclusion criteria encompassed only original research articles published in English between 2010 and 2023 that are thoroughly aligned with the theme of this review article. Updated articles related to the anti-inflammatory activity of bioactive compounds from various marine organisms (seaweed, bryozoan, jellyfish, shellfish and peanut worms) species are summarized and subdivided into subsections similar to those used in our review on anti-inflammatory compounds from echinoderms for the convenience of the reader [15].

## 3. Seaweed as a Marine Source for Anti-Inflammatory Activity

**Figure 1:**
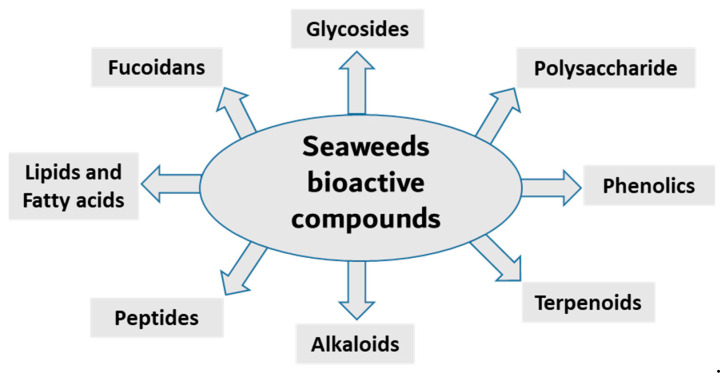
Main classes of seaweed bioactive compounds.

Seaweed is a popular food source rich in bioactive compounds, polysaccharides, fatty acids, peptides, proteins and vitamins. Seaweeds have several potential therapeutic activities, including anti-bacterial, anti-viral, anti-cancer, antioxidant and anti-inflammatory [16,17]. Seaweeds are classified into three groups based on pigment content; for example, Ochrophyta and Phaeophyceae (brown), Chlorophyta (green) and Rhodophyta (red) seaweeds containing fucoxanthin, chlorophyll A, chlorophyll B, phycocyanin and phycoerythrin [18,19,20]. The most diverse are the red seaweeds, with more than 7000 species, followed by the brown and green seaweeds, with approximately 2030 and 600 species, respectively [21]. Researchers have been isolating, purifying and screening the secondary metabolites from these organism for bioactivity in recent decades. Here, we summarize the anti-inflammatory activity of compounds isolated from various seaweed species (Figure 1 and Table 1).

### 3.1. Anti-Inflammatory Phenolic Compounds from Seaweed

**Figure 2:**
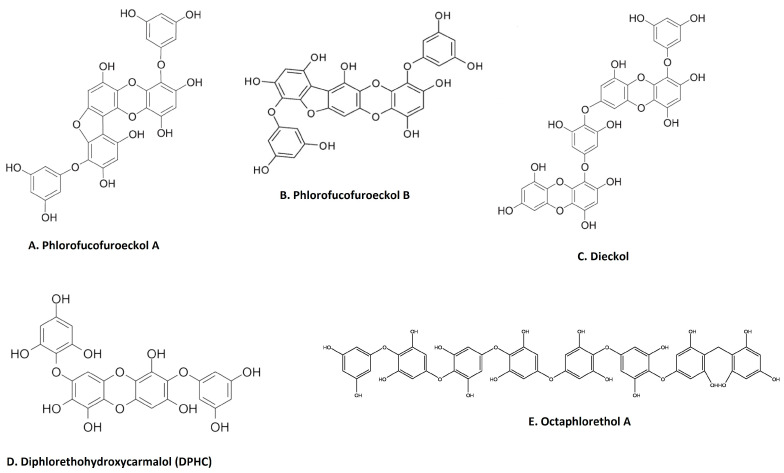
Major anti-inflammatory phenolic compounds isolated from seaweeds (**A**). Phlorofucofuroeckol A from *Eisenia bicyclis*; [22] (**B**). phlorofucofuroeckol B from *Ecklonia stolonifera* [23]; (**C**). dieckol from *Ecklonia cava* [24]; (**D**). diphlorethohydroxycarmalol (DPHC) from *Ishige okamurae* [27]; (**E**) octaphlorethol A from *Ishige foliacea* [55] (structures reproduced with permission from the publisher).

Phenolic compounds are secondary metabolites that are found in many natural extracts and are known for their antioxidant potential. Seaweed is also a rich source of various phenolic compounds, such as flavonoids and tannins, obtained by different extraction methods [49,50,51]. As shown in Figure 2, many categories of phenolic compounds are obtained from seaweeds [51]. The best-studied phenolic compounds are phlorotannins, which exhibit anti-inflammatory effects in addition to other biological activities [52]. A methanol extract of the brown seaweed *Eisenia bicyclis* and its CH_2_Cl_2_ sub-fraction have potent anti-inflammatory effects in LPS-stimulated RAW 264.7 macrophages by inhibiting NO production [22]. Fucosterol, purified through column chromatography from the CH_2_Cl_2_ sub-fraction also decreases NO production via the suppression of iNOS expression [22]. Furthermore, the ethyl acetate sub-fraction of the methanolic extract and chromatographic sub-fractions 1-6 yielded phlorofucofuroeckol A (Figure 2A), eckol, dieckol, phlorofucofuroeckol-A, dioxinodehydroeckol and 7-phloroeckol, which also inhibit NO production in a dose-dependent manner in LPS-stimulated macrophages [22]. Interestingly, similar sub-fractions were extracted from an ethanolic extract and its ethyl acetate sub-fraction from another seaweed, *Ecklonia cava* [53]. These also resulted in significant decreases in the expression of IL1β, IFNγ and interferon stimulatory gene (SGI15) in the olive flounder animal model [53]. The related phlorotannin, phlorofucofuroeckol B (PFFB) (Figure 2B), was isolated and chromatographically purified using NMR from an ethanol extract of the brown algae *Ecklonia stolonifera* [23]. PFFB suppressed the inflammatory response in LPS-stimulated BV2 microglial cells by downregulating the PGE_2_, TNF-α, IL-1β and IL-6 [23]. Furthermore, this study demonstrated that the inhibition of inflammation is via the downregulation of IκB-α/NF-κB mediated by Akt/ERK/JNK pathways [23]. Dieckol (Figure 2C) was isolated from a methanol extract of *Ecklonia cava* powder, with diethyl sub-fractionation, chromatographic purification and NMR characterization [24]. Dieckol decreased NO production via the suppression of iNOS and COX-2 and also inhibited the generation of proinflammatory cytokines IL-1β and TNF-α through suppressing the activation of NF-κB and p38 MAPK in LPS-induced microglial cells [24]. Furthermore, a study using commercially available dieckol revealed the inhibition of carrageenan-triggered inflammation and leukocyte infiltration and reduced pro-inflammatory cytokines (TNF-α, IL-1β, and IL-6) in a mouse model [25]. A recent study demonstrated an increased production of NO and ROS in LPS-treated zebrafish embryos following treatment of an ethyl acetate fraction from *Ecklonia maxima*, which included dieckol [54]. Another compound, diphlorethohydroxycarmalol (DPHC) (Figure 2D), was isolated and purified from the edible brown seaweed *Ishige okamurae.* DPHC suppressed the production of IL-6 via the inhibition of phosphorylation and translocation of NF-κB in LPS-stimulated RAW 264.7 macrophages [27]. Cytokine signaling 1 (SOCS1) suppression functions as a negative feedback regulator of Janus kinase (Jak)-signal transducer and activator of transcription (STAT) signaling [27]. DPHC downregulated STAT5 and upregulated SOCS1 in macrophages [27]. DPHC attenuated several inflammatory symptoms (ear edema, lymph node size, serum IgE level and mast cell infiltration) in an experimental atopic dermatitis-induced inflammatory mouse model [27]. In another study, DPHC purified from the same seaweed also reduced the expression of pro-inflammatory cytokines and suppressed muscle RING-finger protein (MuRF)-1 and muscle atrophy F-box (MAFbx)/atrgoin-1 in LPS-induced RAW 264.7 macrophages [26]. These protein complexes are well known in muscle atrophy via NF-κB and MAPK signaling pathways in TNF-α-stimulated C2C12 myotubes [26]. This study also showed DPHC docking in the TNFα inhibitory site in a simulation [26]. Another interesting compound, octaphlorethol A (Figure 2E), is a phenolic compound isolated from the ethanolic extract, purified from chromatography and further characterized by LC/MS and NMR, belonging to *Ishige foliacea*, which inhibits pro-inflammatory cytokines, MAPK and NF-κB pathways in CpG oligodeoxynucleotides (CpG)-stimulated primary murine bone-marrow-derived macrophages and dendritic cells [28].

### 3.2. Anti-Inflammatory Polysaccharides from Seaweed

**Figure 3:**
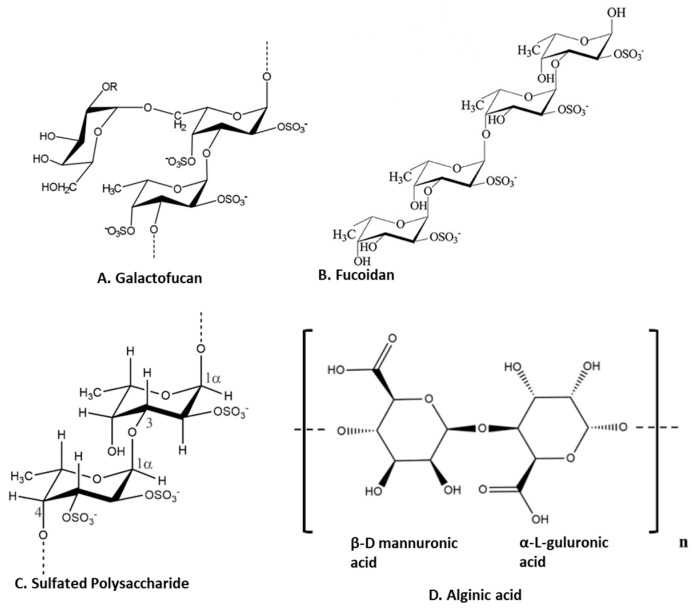
Structure of anti-inflammatory polysaccharides isolated from seaweeds. (**A**). Galactofucans or G-Fucoidan [65]; (**B**). fucoidan found in brown algae [66]; (**C**): sulfated polysaccharides in brown algae [67]; (**D**). alginic acid from brown algae [63] (structures reproduced with permission from the publisher).

Polysaccharides are major components of seaweed that have attracted much attention because of various health benefits [11,56]. Sulfated seaweed polysaccharides show significant anti-inflammatory activity in several inflammatory models [57]. The major compounds are alginic acid and fucoidans, which have various biological effects reflecting their chemical diversity [58] as shown in Figure 3. These compounds are major anti-inflammatory components of seaweed polysaccharides. For example, brown seaweed *Sachharina japonica*-derived fucoidan galactofucan (Figure 3A) demonstrated anti-inflammatory activity by reducing the production of NO and the expression of MAPK (including p38, ENK and JNK) and NF-κB (including p65 and IKKα/IKKβ) signaling pathways in endotoxin-stimulated RAW 264.7 macrophages [29]. Similarly, sulfated fucoidan (Figure 3B) isolated from *Colpomenia sinuosa* prevented oxidative stress and inflammation in the paracetamol-induced hepatic injury and inflammation rat model, as evidenced by suppressing the hepatic levels of thiobarbituric acid reactive substances, NO, iNOS, TNF-α, IL-1β and IL-6 while increasing glutathione and glutathione peroxidase enzyme activity [30]. Fucoidan, extracted from *Fucus vesiculosus*, inhibited LPS-induced inflammatory responses in RAW 264.7 macrophages and zebrafish larvae by suppressing NO and PGE2 secretion via iNOS and COX-2 inhibition as well as reducing the expression and secretion of TNF-α and IL-1β [31]. An ethanol fraction of the hot water extract from the edible seaweed *Laminaria japonica* (which contains abundant fucoidan) suppressed the production of PGE_2_ and expression of MMP-9, COX-2 and pro-inflammatory cytokines in the UV-induced inflammation model of the human keratinocyte (HaCaT) cell line [32]. Interestingly, Nagahwatta et al. purified sulfated fucoidan from the leaves of *Ecklonia maxima* and demonstrated a reduction in proinflammatory cytokines such as PGE2, NO, TNFα IL6 and IL1β in RAW 264.7 macrophages [59]. Furthermore, a recent study revealed that fucoidan had a curative effect mediated by the downregulation of the aryl hydrocarbon receptor and phosphodiesterase 4 in an ulcerative colitis rat model [60]. More recently, sulfated polysaccharides (Figure 3C) isolated from the edible brown seaweed *Sargassum fulvellum* significantly and concentration-dependently decreased the production of the inflammatory mediators NO, PGE2, TNF-α, IL-1β and IL-6, and suppressed the expression of COX-2 and iNOS in LPS-stimulated RAW 264.7 macrophages [33]. Furthermore, these sulfated polysaccharides improve survival and decrease cell death, ROS production and NO levels in LPS-stimulated zebrafish [33]. Another recent study showed the anti-inflammatory effect of a sulfated polysaccharide extracted from *Codium fragile*. This study demonstrated the reduction in PGE2, NO, IL1β, IL6 and TNFα in LPS-induced RAW 264.7 macrophages [61]. The sulfation of seaweed-derived low-molecular-weight fucoidans increases the potency of their anti-inflammatory properties [62]. In contrast, the non-sulfated polysaccharide, alginic acid (Figure 3D), from *Padina boryana*, showed marked anti-inflammatory activity in particulate-matter-stimulated inflammation in human HaCaT immortalized keratinocytes and dermal fibroblasts (HDF) [63]. Alginic acid reduced PGE_2_ and COX-2 and inflammatory cytokines (IL-1β and IL-6) via the suppression of the NF-κB and MAPK pathways [63]. Alginic acid, purified from *Sargassum wightii*, demonstrated anti-inflammatory potential in adjuvant-induced arthritic rats by reducing paw edema and COX, lipoxygenase (LOX) and myeloperoxidase levels [64]. Furthermore, it also reduced the levels of COX-2, IL-6 and TNF-α and inhibited certain key molecular mediators (such as p-p38 MAPK, P-Erk1/2 and P-JNK) of the NF-κB and MAPK pathways in Chinese fine dust (CFD)-treated HaCaT cells [34]. β-Linked polysaccharides, including β-glucans, are known to possess immunomodulatory and anti-proliferative activities. Laminarin, a water-soluble β-glucan isolated from *Grifola frondose*, reduced NO and PGE2 production and suppressed the secretion of pro-inflammatory cytokines via the downregulation of NF-κB in endotoxin-stimulated macrophages [35].

### 3.3. Anti-Inflammatory Terpenoids from Seaweed

**Figure 4:**
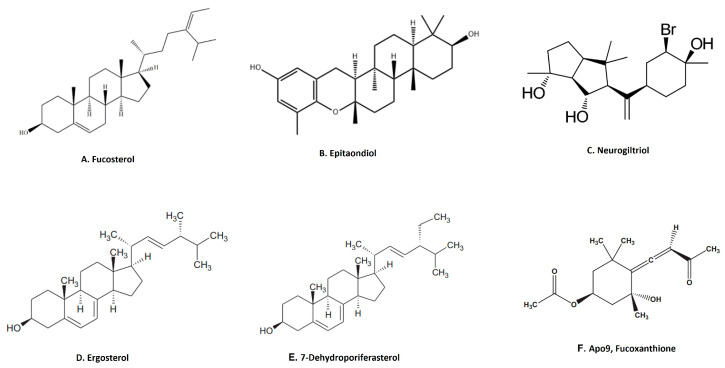
Structure of anti-inflammatory terpenoid extracted from seaweed. (**A**). Fucosterol [36]; (**B**). epitaondiol [37]; (**C**). neurogiltriol [38]; (**D**). ergosterol [39]; (**E**). 7-dehydroporiferasterol [39]; (**F**). Apo9, fucoxanthione [40] (structures reproduced with permission from the publisher).

**Figure 7:**
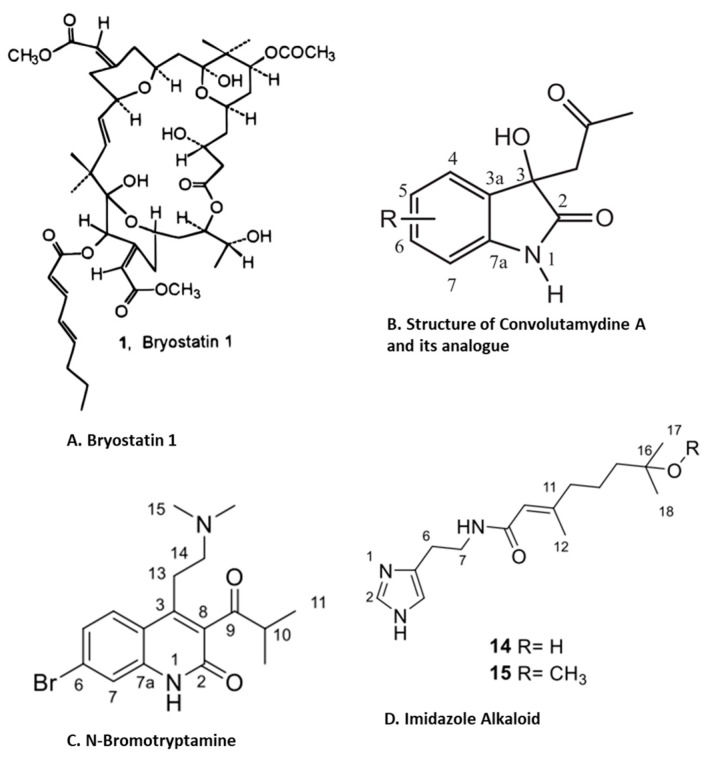
Structure of anti-inflammatory compounds extracted from bryzoans: (**A**). bryostatin 1 [88]; (**B**). convolutamydine A [98]; (**C**). N-bromotryptamine [99]; (**D**). imidazol alkaloids [99] (structures reproduced with permission from the publisher).

Terpenoids are the largest group of natural products with specialized secondary metabolites [68]. These naturally occurring chemical compounds are highly diverse in chemical structure [68]. Although many biological activities of plant-derived terpenoids have been reported, there are several marine-source-based terpenoids that have been isolated and screened for their biological activity in the last few decades [69]. Fucosterol, epitaondiol, neorogioltriol, pacifenol, ergosterol and 7-dehydroporiferasterol, pheophytin A and apo-9′-fucoxanthinone are a few potent anti-inflammatory terpenoids isolated from various species of seaweed [69]. For example, fucosterol (Figure 4A), isolated from *Padina boryana*, reduced particulate matter (PM)-induced inflammation in macrophages by modulating the NF-κB and MAPK pathways [36]. It significantly suppressed the expression of inflammatory mediators such as iNOS, COX-2, pro-inflammatory cytokines and PGE2 [36]. Epitaondiol, isolated from various seaweeds, inhibited COX pathway activity. For example, epitaondiol (Figure 4B), isolated from *Stypopodium flabelliforme*, inhibited the production of phospholipase A2 and eicosanoids (LTB4 and TXB2) and modulated the COX pathway in the in vitro isolated human neutrophils and the 12-O-Tetradecanoylphorbol-13-acetate (TPA)-induced mouse ear oedema model [37]. Neorogioltriol (Figure 4C), and two related diterpene neorogioldiol and O11,15-cyclo-14-bromo-14,15-dihydrorogiol-3,11-diol, were isolated from a red algae *Laurencia glandulifera* [38]. These compounds exhibit anti-inflammatory effects in M2-type macrophages via an increased expression of arginase1, MRC1, IRAK-M and the transcription factor C/EBPβ [38]. The anti-inflammatory activity of neorogioldiol and O11,15-cyclo-14-bromo-14,15-dihydrorogiol-3,11-diol was also demonstrated in a DSS-induced colitis mice model where they reduced tissue damage and pro-inflammatory cytokine production in vivo. Ergosterol (Figure 4D) and 7-dehydroporiferasterol (Figure 4E) derivatives, isolated from *Dunaliella tertiolecta*, suppressed pro-inflammatory cytokines in sheep peripheral blood mononuclear cells stimulated with LPS [39]. Apo-9-fucoxanthinone (AF), isolated from *Sargassum muticum*, showed potent anti-inflammatory activity in LPS-stimulated macrophages by suppressing the mRNA expression of several inflammatory mediators, such as iNOS, COX-2 and pro-inflammatory cytokines [40]. These effects of AF are mainly due to the modulation of NF-κB and MPAK signaling pathways [40].

### 3.4. Anti-Inflammatory Proteins and Peptides from Seaweed

Lectins, isolated from seaweed, are glycoproteins involved in cellular adhesion. Some of these lectins have anti-inflammatory activity. A 30 kDa lectin derived from *Amansia multifida* has anti-inflammatory effects, reducing edema formation in a paw edema model, leukocyte migration and oxidative stress in a carrageenan-induced peritonitis model and proinflammatory cytokine (IL-1β and TNF-α) expression in a carrageenan-induced rat paw edema model [41]. Another study demonstrated the anti-inflammatory effects of a 9kDa lectin isolated from *Bryothamnion triquetrum* [42]. This lectin inhibited the production of proinflammatory cytokines involved in the migration of neutrophils in a carrageenan-induced peritonitis mice model [42]. These findings suggest that the anti-inflammatory effects of this lectin are mediated by the inhibition of leukocyte recruitment [42]. In another study, a mucin-binding hololectin isolated from red marine algae demonstrated anti-nociceptive and anti-inflammatory responses, reducing abdominal writhing and the paw-licking time in carrageenan-induced peritonitis and paw edema models [70]. Hololectin caused a similar inhibition of neutrophil migration in a peritonitis model and also suppressed paw edema, which was induced by carrageenan, dextran or serotonin [70]. A big anti-inflammatory effect (23–44 kDa) from the green seaweed *Caulerpa cupressoides* revealed a decrease in carrageenan-induced rat paw edema and neutrophil infiltration via a reduction in the expression of IL-1, IL-6, TNF-α and COX-2 [71]. Lectins from red algae and their other potential biomedical applications have been previously summarized in the review [72]. Only a limited number of papers reporting the anti-inflammatory activity of lectins from green seaweeds have been published. However, lectins from red and green algae have distinct structures [73]. A recent review documented the structural diversity among various algal species [73]. The authors classified the lectins from red and brown algae as *Oscillatoria agardhii* agglutinin homolog (OAAH) and the green seaweed as *Galanthus nivalis* agglutinin (GNA) [73]. A study on the structural features of N-glycans of seaweed glycoproteins was performed on 15 economically important seaweeds (12 red and brown seaweeds, 2 green seaweeds and 1 sea grass) [74]. Interestingly, this study revealed the absence of typical plant-based lectins with a complex structure of N-glycans (β1-2 xylosyl and α 1-3 fucosyl) in algae and seagrass [74]. Moreover, the study showed the absence of high manose-type N-glycan (M5–M9) in the green seaweed *Ulva* pertusa [74]. A study on peptide fractions of approximately 2160 kDa from green seaweed *Ulva* spp. showed it exerting immunomodulatory actions in vitro, consistent with an anti-inflammatory effect depending on Toll-like receptor 4 (TLR4) and the NFκB/p38/JNK pathway [43].

### 3.5. Anti-Inflammatory Alkaloids from Seaweed

**Figure 5:**
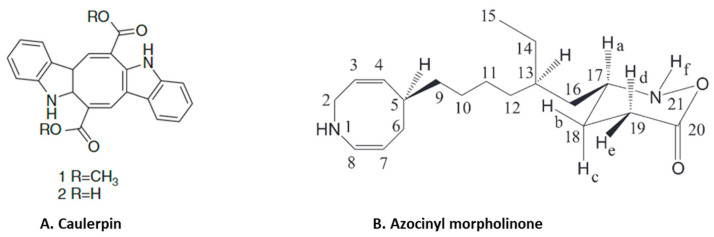
Structure of anti-inflammatory alkaloids: (**A**). caulerpin [80]; (**B**). azocinyl morpholinone [45] (structures reproduced with permission from the publisher).

Anti-inflammatory alkaloids are commonly found in plants, but there are limited studies identifying and characterizing the activity of such compounds from seaweeds [75]. Only limited studies have investigated the anti-inflammatory effects of seaweed alkaloids [75]. Caulerpin (Figure 5A) is an indole alkaloid with anti-inflammatory activity isolated from several different species of seaweed [76]. This compound is one of the main products in a study of the anti-inflammatory effect of methanolic extracts from *Caulerpa racemosa* [76]. Caulerpin reduced inflammation in DSS-induced ulcerative colitis, where it suppressed inflammatory infiltration and reduced the levels of colonic proinflammatory cytokines (IL-6, IL-17, TNF-α and IFN-γ) while increasing the expression of the anti-inflammatory cytokine IL-10 [44]. These effects of caulerpin were accompanied by a reduction in the expression of NF-κB p65, suggesting that the anti-inflammatory effects are mediated by blocking the activation of NF-κB [44]. Anti-inflammatory alkaloids forming red algae of the genus *Gracilaria* have been identified [77]. An aqueous extract containing polyphenols, flavonoids and ascorbic acid from *Gracilaria tenuistipitata* had anti-inflammatory activity in a hepatitis C virus model [78]. Treatment with this extract inhibited COX-2 activity, the synthesis of PGE_2,_ nuclear translocation of NF-κB p65 and expression of TNF-α, IL-1β and iNOS in HCV-infected cells [78]. Fractions of a methanol extract, analyzed by mass spectroscopy, from *Gracilaria changii* reduced the expression of TNF-α and IL-6 in phorbol 12-myristate 13-acetate (PMA)-stimulated U937 cells [79]. An anti-inflammatory alkaloid, azocinyl morpholinone (Figure 5B), isolated from *Gracilaria opuntia* selectively inhibited COX-2 and 5-LOX activity and thereby reduced inflammation in a murine carrageenan-induced paw edema model [45].

### 3.6. Other Anti-Inflammatory Compounds from Seaweed

**Figure 6:**
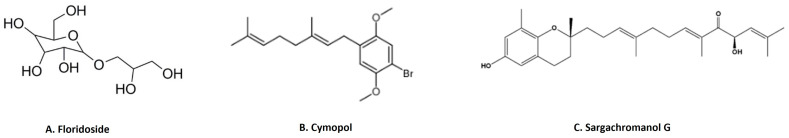
Structure of few important anti-inflammatory compounds from seaweed: (**A**). floridoside from *Laelia undulata* [84]; (**B**). cymopol from *Cymopolia barbata* [48]; (**C**). sargachromanol G from *Sargassum siliquastrum* [81] (structures reproduced with permission from the publisher).

3-Hydroxy-4,7-megastigmadien-9-one isolated from *Ulva pertusa* markedly inhibits interleukin (IL)-12 p40, IL-6 and TNF-α cytokine production [46]. The compound exhibits anti-inflammatory activity through the inhibition of MAPK by the dephosphorylation of ERK1/2, JNK12 and p38, as well as inhibition of the NF-κB pathway by the dephosphorylation of IκBα in CpG-stimulated bone-marrow-derived dendritic cells [46]. This study also demonstrated that the compound downregulates the TLR9 promoter activity through AP1 and NF-κB [46]. Floridoside (Figure 6A) is another anti-inflammatory compound derived from Laelia undulata, and inhibits the production of NO and ROS in LPS-stimulated BV2 microglial cells [47]. This study demonstrated the floridoside-mediated downregulation of iNOS and COX-2 by blocking the phosphorylation of p38 and ERK in LPS-stimulated BV-2 cells [47]. A non-polar extract from *Cymopolia barbata* and its primary active component cymopol (Figure 6B) were analyzed for their anti-inflammatory properties [48]. Purified cymopol upregulates the Nrf2 transcription activity and reduces the expression of proinflammatory genes such as iNOS, COX2, PGE2 and Nqo1 with an expected reduction of NO in macrophages and mouse embryonic fibroblasts in an Nrf2-dependent manner [48]. Cymopol-containing extracts also attenuate neutrophil migration in a zebrafish tail wound model [48] and reduce DSS-induced colitis, as measured by fecal lipocalin concentration [48]. Further analysis of DSS-induced mice treated with a cymopol fraction and the non-polar extract through RNA-seq revealed the enrichment of mucosal-associated microbiome genera [48]. Another compound sargachromanol G (Figure 6C), isolated by chromatography from *Sargassum siliquastrum*, reduces the expression of pro-inflammatory cytokines (TNF-α, IL-1β and IL-6) and also suppresses inflammatory markers such as NO and PGE2 production via the inhibition of iNOS and COX-2 in RAW 264.7 cells [81]. A study of sargachromanol G on osteoclast differentiation revealed the inhibition of the receptor activator of the NF-κB ligand (RANKL)-induced activation of NF-κB by suppressing RANKL-mediated IκB-α degradation [82]. The study further explored the mechanism of action of sargachromanol G and demonstrated the inhibition of mitogen-activated protein kinases (p38, JNK and ERK) in RANKL-stimulated RAW 264.7 macrophages [82]. A recent study showed anti-inflammatory activity of an ethanol extract of *Sargassum siliquastrum* in RAW 264.7 cells by the inhibition of NO and proinflammatory cytokines such as TNFα and IL-6 [83]. Further studies are needed to explore these anti-inflammatory active agents responsible.

## 4. Bryozoans as Marine Source for Anti-Inflammatory Activity

Marine bryozoans are a diverse group of invertebrates, inhabiting everywhere from intertidal waters to deep seas in both tropical and polar regions [85]. Diverse species of bryozoans are found in the North Sea, the United Kingdom, the northern Mediterranean and Adriatic, North Pacific around Japan, New Zealand and Antarctica [86]. Bryozoans produce bioactive compounds that are an important source of bioactive compounds important for their development as drugs [87]. They have attracted attention due to the discovery of the remarkable anti-neoplastic activity of bryostatins [88]. A host of secondary metabolites, including alkaloids, sphingolipids, sterols, macrocyclic lactones, other tetracyclic terpenoid lactones and sulfur-containing aromatic compounds, have been isolated from bryozoans [87]. However, in our search for the anti-inflammatory activity of bioactive compounds isolated from marine bryozoans for this review, we found only five papers (Table 2). Two major chemical classes, macrocyclic lactones (bryostatin-1) and alkaloids (convolutamydine A and bromotryptamine) isolated from various species of bryozoan, have been evaluated for anti-inflammatory activity [88]. Bryostatins are macrocyclic lactones first isolated and characterized from Bugula neritina [88]. In a study, around 21 bryostatins were isolated and studied; however, bryostatin (bryos-1) (Figure 7A) has received the most attention from the scientific community as a therapeutic option in several inflammatory diseases [89]. For example, bryos1 has been extensively evaluated as a drug of choice for multiple sclerosis (MS) [90]. The characteristic etiology of MS involves inflammation and myelin damage, which involves multiple steps, including the overexpression of pro-inflammatory cytokines, induction of oxidative stress, overexpression and high levels of activity of matrix metalloproteases (MMPs) and loss of the blood–brain barrier (BBB), which ultimately lead to the immune-mediated destruction of neuronal myelin damage and neuronal degeneration [90]. Bryos-1 was reviewed as a target of multiple MMPs, including MMP 1, 3, 9, 10 and 11, in various models [91]. Moreover, the downregulation of MMP-9 expression and its activation by Bryos-1 reduced hemorrhagic transformation followed by ischemia–reperfusion injuries in an aged female rats model [92]. Bryos-1 also demonstrated an increase in the IL-4-induced expression of the anti-inflammatory marker arginase-1, as well as tissue repair promoting the M2 macrophage phenotype in murine peritoneal macrophages [93]. Similar to its effect on dendritic cells, bryos-1 suppresses the production of pro-inflammatory cytokines (IL-12 and IL-6) while increasing the production of the anti-inflammatory cytokine (IL-10) [94]. A recent study demonstrated the anti-neuroinflammatory effects of the bryos-1 analogue, bryologs, both in vitro and in vivo [95]. In contrast to anti-inflammatory activity, bryos-1 enhances proinflammatory effects by increasing CCL2, IL-10, TNFα, etc., in the context of the analysis of bryos-1 as a latency-reversing agent in HIV pathophysiology [96].

Similarly, bryos-1 also demonstrated to be effectively anti-inflammatory during carcinogenesis. For example, bryos-1 downregulated COX-2 mRNA expression in the mucosa of 1,2-dimethylhydrazine (DMH)-induced colorectal carcinogenesis in rats [93]. Furthermore, it induces the anti-inflammatory phenotype of macrophages and antigen-presenting cells (APCs) [93]. It upregulates the CD86 protein, which is required for the activation of T cells upon their binding to APCs in bone-marrow-derived dendritic cells (BMDCs) [93]. Moreover, recently, the Food and Drug Administration (FDA) approved bryos-1 as an orphan drug in combination therapy with paclitaxel for the treatment of esophageal carcinoma [97].

Convolutamydine A (Figure 7B), an oxindole alkaloid isolated from marine bryozoan species *Amathia convoluta*, was evaluated for anti-inflammatory activity. Convolutamydine A and its two analogues (ISA003 and ISA147) inhibit the formalin-induced licking response at doses as low as 10 µg/kg. These compounds also inhibit leukocyte migration and the production of NO, PGE2 and inflammatory cytokines (IL-6 and TNF-α) in the subcutaneous air pouch (SAP) model of carrageenan-induced inflammation. Furthermore, convolutamydine A and the two analogues reduce NO and PGE2 production by the downregulation of COX-2 and iNOS in cultured macrophages [98]. Recently, Di et.al isolated 13 new bromotryptamines (Figure 7C) and two new imidazole alkaloids (Figure 7D) from the bryozoan Flustra foliacea and evaluated their in vitro anti-inflammatory activity [99]. Several of these newly isolated alkaloids, flustramine Q, flustramine S, flustramine U, Nα-methyldeformylflustrabromine, 6-bromo-N, N-dimethyltryptamine, 6-bromoindole-3-carbaldehyde and deformylflustrabromine B, decreased the dendritic cell secretion of the pro-inflammatory cytokine IL-12 p40 while flustrimidazole A and flustramine T increased the secretion of the anti-inflammatory cytokine IL-10 in monocyte-derived dendritic cells [99].

**Table 2:** Anti-inflammatory substances derived from bryozoans.

Species	Bioactive Compounds/ Extracts/ Purified Compound	Model Controls	Anti-inflammatory Activity	Ref.
*Bugula* *neritina*	Bryostatin-1 purified and procured from Sigma.	DMH-induced colorectal carcinogenesis in rat as positive control and infection with *Syphacia muris*.	Downregulates COX-2 mRNA expression in colorectal mucosa at 5 µg/kg body weight for 4 weeks.	[93]
	Purified bryostatin-1 procured from Tocris.	Stimulated macrophages, antigen-presenting cells and bone-marrow-derived dendritic cells.	Activates T cell via upregulation of CD86. Increases IL-4-induced expression of arginase-1 and increases M2 macrophages. Suppresses production of pro-inflammatory cytokines (IL-12 and IL-6) while increasing the production of anti-inflammatory cytokine (IL-10) at concentration ranges from 20 to 200 nM.	[94]
	Purified bryostatin-1.	Acute cerebral ischemia in aged rat model. R-tPA is used as a positive control.	Suppresses MMP-9 by upregulating PKCε at 2.5 mg/kg body weight.	[92]
*Amathia* *convolute*	Isatin converted by acetome and dienthlamine at room temperature to convolutamydine A.	Carrageenan-induced inflammation model. LPS-stimulated macrophages.	Suppresses leucocyte migration, reduces the production of NO and PGE2 by downregulating iNOS and COX-2 and decreases IL-6 and TNF-α production at 0.1 to 10 mg/kg body weight.	[98]
*Flustra* *foliacea*	Bromotryptamine and imidazole alkaloids purified through chromatography and characterized by NMR.	Monocyte-derived dendritic cells.	Decreases pro-inflammatory cytokine IL-12p40 and increases secretion of the anti-inflammatory cytokine IL-10 at 10 µg/mL.	[99]

## 5. Anti-Inflammatory Compounds from Jellyfish

**Figure 8:**
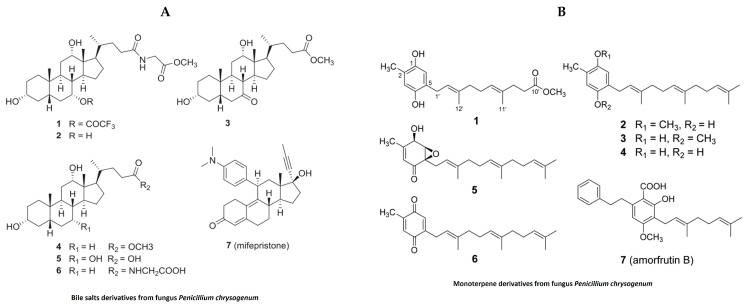
Structure of anti-inflammatory bile salts and monoterpenes derivative from jellyfish: (**A**). bile salts derivatives [102]; (**B**). monoterpene derivatives from jellyfish fungus penicillium chrysogenum [103] (structures reproduced with permission from the publisher).

In the past, jellyfish have elicited unpleasant sentiments in European culture, while they are regarded as a valuable source of bioactive substances and are utilized in traditional food and medicine on the Asian continent [100]. In addition to their nutritional and medicinal benefits in the Chinese pharmacopeia, jellyfish have recently been designated as a novel food in Western countries due to their unexploited source of important nutrients, novel bioactive metabolites and lead compounds [100]. In the last decade, extracts derived from different species of jellyfish have been evaluated for pharmacological properties. Despite this, our literature search for anti-inflammatory activities of bioactive compounds isolated from jellyfish produced only limited results (Table 3). Polysaccharides isolated from the jellyfish *Rhopilema esculentum* modulate oxidative stress and inflammation in the DSS-induced colitis model in mice [101]. These polysaccharides reduced myeloperoxidase (MPO) activity, levels of pro-inflammatory cytokines and NO levels in mice with colitis [101]. The polysaccharides also downregulate the NF-κB signaling pathway in colonic tissue, as evident by reducing the phosphorylation of p65 and IKB [101]. Bile salt (Figure 8A) derivatives isolated from a fungus (*Penicillium chrysogenum* J08NF-4), which specifically grows on the jellyfish *Nemopilema nomurai*, demonstrated a suppression of the NO level in LPS-stimulated RAW 264.7 macrophages [102]. Interestingly, the study also shows the downregulation of many proinflammatory molecules, including iNOS and TNF-α [102]. Molecular docking studies revealed that one these bile salt derivates, a bile salt trifluoroacetate, has a ligand-binding domain for the PPARγ receptor that activates PPARγ and suppresses the phosphorylation of the NF-κB p65 subunit, leading to the downregulation of pro-inflammatory mediators [102]. Monoterpene derivatives (Figure 8B) isolated from the same fungus also exhibit PPARγ receptor agonistic activity in molecular docking studies and also suppress PPARγ-mediated inflammatory markers in Ac2F liver cells [103]. A recent study showed anti-inflammatory properties of collagen hydrolysate from jellyfish in a high-fat diet mice model. This study demonstrated the reduction in proinflammatory cytokines such as TNFβ, IL1β and IL6 [104].

## 6. Anti-Inflammatory Compounds from Shellfish

Shellfish constitute a major seafood component [105]. Common subgroups of shellfish are crustaceans, bivalves (mollusks), gastropods (univalves) and cephalopods [105]. Important crustaceans are prawns, shrimp, lobster, crab and krill. Bivalves (two shells) include clam, mussel, oyster and scallops [105]. Univalves (single shell) include cockle, whelk, limpet, abalone and snail. While cephalopods lack an external shell, they are also considered to be shellfish, and this group includes octopus, cuttlefish and squid [105]. Shellfish foods have a high nutrition value, as shown by a high protein digestibility and corrected amino acid score [105]. They are also rich in Omega 3 fatty acids, carotenoids and vitamins [105]. In addition to their high nutritive value, shellfish are also considered as a valuable source of nutraceutical compounds [105]. Here, we summarize the anti-inflammatory activities of bioactive compounds isolated from shellfish, including mussels, clams and other mollusks [106] (Table 3). A novel peptide (EGLLGDVF) isolated and characterized from the green mussel *Perna viridis* has potent anti-inflammatory activity, inhibiting the production of pro-inflammatory cytokines, reducing the activation of NO and COX-2 and downregulating iNOS and COX-2 expression in LPS-stimulated RAW 264.7 macrophages [107]. Similarly, high-molecular-weight peptides isolated from the blue mussel *Mytilus edulis* exhibited marked anti-inflammatory activity, modulating NF-κB and MAPK pathways in LPS-stimulated RAW 264.7 macrophages [108]. *Mytilus coruscus* is a Korean hard-shelled mussel that is heavily exploited for food. Various bioactive compounds (peptides and lipids) from this mussel have been isolated and tested for their anti-inflammatory potential. A novel peptide consisting of 10 amino acid residues (GVSLLQQFFL) was isolated, which effectively blocks NO production in LPS-stimulated RAW 264.7 macrophages [109]. A high-molecular-weight α-d-Glucan named MP-A, also isolated from this hard-shelled mussel, exerts anti-inflammatory activity in LPS-stimulated THP1 cells by inhibiting cytokine production and suppressing iNOS and COX-2 expression via TLR4/NF-κB/MAPK pathway inhibition [110]. A lipid extract from this hard- shelled mussel increases the production of the anti-inflammatory cytokine (IL-10) and suppresses pro-inflammatory cytokines via the downregulation of toll-like receptor (TLR-4) signaling in a lipopolysaccharide (LPS)-challenged mouse model [111]. The lipid extract of this hard-shelled mussel also showed anti-inflammatory activity in adjuvant-induced (AIA) and collagen-induced arthritis (CIA) in rat models, suppressing markers of inflammation such as leukotriene B4 (LTB4), prostaglandin E2 (PGE2) and thromboxane B2 (TXB2) in the ankle joint synovial fluid of treated rats [112]. Green-lipped mussels *(Perna canaliculus*) are heavily cultivated in New Zealand. The oil fractions Lyprinol and Cadalmin from green-lipped mussels are promoted as functional foods with numerous health benefits [113]. These lipid fractions markedly suppressed iNOS and COX-2 and downregulated inflammatory cytokine genes via the inhibition of the NF-κB/MAPK pathways in LPS-stimulated RAW 264.7 murine macrophages [113]. A clinical trial of Lyprinol (PSCOl524) showed a beneficial effect on the bronchoconstriction of 24 asthma patients [114]. Recently, an interesting research article isolated a peptide from the Asiatic hard clam *Meretrix meretrix* and tested its anti-inflammatory potential [115]. The isolated 6 amino acid peptide (HKGQCC) significantly inhibited NO, COX-2, TNF-α and IL-1β in LPS-stimulated RAW 264.7 cells [115]. The in vitro digestion of this peptide resulted in the generation of a tetrapeptide (GQCC) that further downregulates inflammatory gene expression in an LPS-stimulated zebrafish model [115]. The abalone (*Haliotis discus hannai*) is a large marine gastropod mollusk that is a valuable seafood product [116]. An 11-amino-acid peptide (EMDEAQDPSEW) isolated from abalone has potent anti-inflammatory activity by inhibiting MAPK and NF-κB signaling pathways in human fibrosarcoma (HT1080) cells [116]. Phenoloxidase (PO), purified from the hemolymph of the dietary shellfish *Haliotis discus hannai*, has anti-inflammatory effects on LPS-induced HT 29, reducing levels of pro-inflammatory cytokines, including IL4, IL5 and IFNγ and PGE_2_ [117]. In a recent study, three lipid fractions (neutral lipids, glycolipids and phospholipids) from the eggs of a common dietary shellfish *Ammodytes personates* downregulated proinflammatory cytokines in an LPS-induced RAW 264.7 macrophage model. The downregulation of NFkB signaling through MAPK signaling pathways was also seen [118].

**Table 3:** Anti-inflammatory substances derived from jellyfish and shellfish.

Marine Source	Species	Bioactive Compounds/ Extracts/Purified	Model	Anti-Inflammatory Activity	Ref.
Jellyfish	*Rhopilema esculentum*	Skin polysaccharide and monosaccharide composition analysis.	DDS-induced colitis mice model.	Reduces MPO activity, pro-inflammatory cytokines and NO levels. Downregulates NF-κB at 50 and 100 mg/kg bodyweight.	[101]
	*Nemopilema nomurai* *Penicillium chrysogenum* J08NF-4	Bile acid derivates and monoterpene purified from fungal strain through HPLC.	LPS-stimulated RAW 264.7 cells.	Suppresses production of cytokines. Activates PPARγ-mediated NF-κB inhibition at 10 to 50 µM.	[102,103]
Shellfish	*Perna viridis* (Green mussel)	Peptide (EGLLGDVF) purified of about 850 Da.	LPS-stimulated RAW 264.7 cells.	Suppresses pro-inflammatory cytokines. Downregulates iNOS and COX-2.	[107]
	*Mytilus edulis* (Blue mussel)	Peptide fraction obtained from enzyme hydrolysate.	LPS-stimulated RAW 264.7 macrophages.	Inhibits the NF-κB/MPAK signaling pathway at concentration ranges from 50 to 200µg/mL.	[108]
	*Mytilus coruscus* (Korean hard-shelled mussel)	Peptide (GVSLLGGPPL) purified and characterized from enzyme hydrolysate.	LPS-stimulated RAW 264.7 cells.	Reduces NO production.	[109]
	*Mytilus coruscus* (Korean hard-shelled mussel)	Lipid extract (HMLE).	Adjuvant-induced and collagen-induced arthritis.	Suppresses markers of inflammation such as LTB4, PGE2 and TXB2 in ankle joint synovial fluid.	[112]
	*Mytilus coruscus* (Korean hard-shelled mussel)	Lipid extract (HMLE) and analyzed by gas chromatography.	LPS-challenged MS Dowley rats model using adjuvant-induced arthritis as positive control.	Increases the production of IL-10 and suppresses IL-1, IL-6 and TNF-α via downregulation of TLR-4 signaling pathways at 100mg/kg body weight.	[111]
	*Mytilus coruscus* (Korean hard-shelled mussel)	α-d-Glucan (MP-A) purified by chromatography obtained.	THP1 differentiated by PMA and then stimulated by LPS.	Inhibits cytokine production, downregulates iNOS and COX-2 and inhibits TLR4/NF-κB/MAPK pathway at 10 to 200 µg/mL.	[110]
	*Perna canaliculus* (Green-lipped mussel)	Oil fraction (GLMO) purified form obtained.	LPS-stimulated RAW 264.7 cells.	Inhibits iNOS and COX-2. Downregulates cytokine gene expression via NF-κB/MAPK pathway at 50 to 300 µg/mL.	[113]
	*Meretrix meretrix* (Clam)	Peptide (HK and GQCC) purified from enzyme hydrolysate through HPLC.	Human blood in vitro assays, LPS-stimulated RAW 264.7 cells and zebrafish.	Inhibits NO, NO, TNF-α, IL-1β and COX-2 at 50 to 250 µg/mL.	[115]
	*Haliotis discus hannai* (Mollusk)	Peptide purified and characterized of approximately 1234.41 Da.	PMA-challenged human fibrosarcoma (HT1080) cells.	Inhibits MMPs expression via modulation of MAPK and NF-κB pathway at 50 and 100 µM.	[116]

## 7. Anti-Inflammatory Compounds Derived from Peanut Worms

Peanut worms are a class of unsegmented marine annelids commonly found in marine benthic ecosystems and used as a functional food in many places, including South China and the Philippines [119]. Many compounds with anti-inflammatory activity have been identified in peanut worm species [119] (Table 4). For example, a study reported the anti-inflammatory activity of an aqueous extract from the body wall of *Sipunculus nudus* in different rat and mouse models such as a carrageenan-induced rat paw oedema model, Dextran-induced rat paw oedema model, cotton-pellet-induced chronic inflammation granuloma rat model, carrageenan-induced peritonitis mouse model, xylene-induced ear oedema model and acetic-acid-induced vascular permeability mice model [120]. In another study, the researchers established the presence of hydrophobic amino-acid-rich peptides within the collagen fraction of the peanut worm *Sipunculus nudus* [121]. They demonstrated the anti-inflammatory function of a novel peptide mediated through a reduction of nitric oxide (NO) in LPS-stimulated RAW 264.7 macrophages [121]. Furthermore, the authors confirmed the inhibitory effect of peptides on the mRNA expression of iNOS, TNFα, IL6 and COX-2 [121]. Another research group from China identified a similar peptide fraction of peanut worm powder [122]. They demonstrated the peptide-mediated induction of skin wound healing function in mice within four days of a wound and complete healing within 28 days [122]. Furthermore, they established that the wound-healing property of these peptides is mediated by a reduction in the expression of proinflammatory cytokines such as TNFα and IL1β [122]. The peptides also had anti-scar activity due to the reduction in TGFβ1 mRNA [122]. Another important species of peanut worm is *Phascolosoma esculenta*, commonly used as a functional food in China [123]. An oligosaccharide fraction extracted from this species by enzymatic hydrolysis has anti-inflammatory effects in an *Escherichia coli*-induced sepsis mouse model via a reduction in the expression of the proinflammatory cytokines TNFα and IL1β and an enhanced expression of IL10 [123].

## 8. Conclusions and Future Perspective

Marine biodiversity provides a promising resource for pharmacology. Seaweeds are a major source of bioactive compounds with potent anti-inflammatory effects. Major categories of seaweed-derived bioactive compounds include phenolic compounds, polysaccharides, glycoproteins, polypeptides, terpenoids and alkaloids. These compounds strongly inhibit lipoxygenases and cyclooxygenases and decrease the ROS and NOS levels. Moreover, these compounds also downregulate proinflammatory cytokines such as IL6 and TNFα through the inhibition of NF-ƙB pathways. Bryozoans provide another rich source of marine pharmaceutical compounds. Compounds from these organisms include bryostatin-1, which exhibits strong anti-inflammatory activity. Jellyfish and shellfish provide a major marine resource widely used for food and as nutraceuticals. However, some of them have been investigated and this has revealed multiple bioactive compounds, including bile acids, monoterpenes, lipids and peptides, with anti-inflammatory activity. This review covers the anti-inflammatory activity of bioactive compounds from seaweed, bryozoans, jellyfish and shellfish. It complements our previous review of anti-inflammatory compounds from echinoderms. These reviews can serve as a source of summarized information for standardizing marine sources and developing analytical methods to quantify anti-inflammatory compounds. By integrating these approaches, we can develop safer and more effective anti-inflammatory agents from marine sources. New technologies and close collaborations between institutional and industrial investigators are crucial for the development of marine-derived bioactive compounds to be successful as novel therapeutics in treating or preventing chronic diseases.

## Figures and Tables

**Table 1:** Anti-inflammatory bioactive substances derived from seaweed.

Species	Bioactive Compounds/ Extracts/ Purified Compounds	Model	Anti-Inflammatory Activity	Ref.
*Eisenia bicyclis*	Phlorofucofuroeckol A (compound purified by HPLC and characterized by NMR).	Lipopolysaccharide (LPS)-stimulated RAW 264.7 macrophages.	Suppresses LPS-induced nitric oxide (NO) production at 10 µg/mL. In this study, 10 µM AMT 2-amino-5,6-dihydro-6-methyl-4H-1,3-thiazine was used as a positive control.	[22]
*Ecklonia* *stolonifera*	Phlorofucofuroeckol B (compound purified by HPLC and characterized by NMR).	LPS-stimulated microglial cells.	Inhibits secretion of tumor necrosis factor (TNF) -α, interleukin (IL)-1β and IL-6, downregulates the transcription of cycloxygenase (COX)-2 and iNOS synthase, inhibits IκB-α/NF-κB and Akt/ERK/JNK pathways at concentrations of 10 µM–40 µM.	[23]
*Ecklonia* *cava*	Dieckol (compound purified by HPLC and characterized by NMR).	LPS-stimulated microglial cells.	Suppresses LPS-induced mRNA expression of inflammatory mediators COX-2 and iNOS and NO production at concentrations 50 µg/mL to 300 µg/mL. Inhibits IL-1β and TNFα production. Reduces mRNA expression of NF-κB and p38 MAPK.	[24]
*Ecklonia* *cava*	Dieckol (commercial dieckol is used in this study).	Carrageenan-triggered inflammation in mice.	Dieckol inhibits carrageenan-triggered inflammation, leukocyte infiltration and formation of pro-inflammatory regulators such as TNFα, IL1β, IL6, etc. at dosages from 5 mg/kg–20 mg/kg bodyweight.	[25]
*Ishige* * okamurae*	Diphloretho- hydroxycarmalol purified from aqueous methanol extract through HPLC and characterized by NMR.	LPS-induced RAW 264.7 cells and TNF-α-stimulated C2C12 myotubes.	Downregulates mRNA expression of pro-inflammatory cytokines, reduces NO production and reduces protein expression of NF-κB and p38 MAPK at concentration ranges from 6 to 200 µg/mL.	[26]
*Ishige* *okamurae*	Diphloretho- hydroxycarmalol purified from aqueous methanol extract through HPLC and characterized by NMR.	LPS-stimulated RAW 264.7 macrophages.	Downregulates IκB-α and NF-κB protein expression and inhibits IL-6 production by downregulating STAT5 activation and SOCS1 augmentation at concentration ranges from 12.5 to 100 µM.	[27]
*Ishige* *foliacea*	Octaphlorethol A purified from aqueous ethanolic extract and characterized by LC/MS and NMR.	CpG- stimulated C57BL/6 mice bone-marrow-derived macrophages and bone-marrow-derived dendritic cells.	Exhibits anti-inflammatory activity by octaphlorethol A by transcriptional regulation of NF-κB through MAPK at concentration ranges from 1.5 to 50 µM.	[28]
*Saccharina* *japonica*	Fucoidan purified from ethanolic extract by dialysis and HPLC.	LPS-stimulated RAW 264.7 macrophages and LPS-induced zebrafish embryonic cells.	Reduces the production of NO and downregulates the expression of MAPK (including p38, ENK and JNK) and NF-κB (including p65 and IκBα/ IκBβ) signaling pathways at concentration ranges from 12.5 to 50 µg/mL.	[29]
*Colpomenia* *sinuosa*	Fucoidan purified from ethanol:formaldehyde:water solvent system through acid extraction and purification.	In vitro NO scavenging assay and RBC hemolysis and PCM-induced hepatic injury in rat.	Prevents paracetamol-induced hepatic oxidative stress and decreases NO, iNOS, TNFα, IL-1β and IL-6 in liver tissue at a concentration of 10 to 50 µg/mL.	[30]
*Fucus* *vesiculosus*	Fucoidan from *Fucus vesiculosus* purchased from Sigma.	LPS-stimulated RAW 264.7 macrophages and LPS-induced zebrafish embryonic cells.	Decreases secretion of NO, prostaglandin (PG) E2, TNFα and IL-1β at concentration ranges from 0.5 to 10 µg/mL.	[31]
*Fucus vesiculosus*	Fucoidan crude hot water extract.	UV-induced inflammation in HaCaT cells.	Decreases production of NO, PGE2, IL-1β and TNFα, and inhibits NF-κB, Akt, ERK, p38 MAPK and JNK pathways regulated by nc886-PKR.	[32]
*Sargassum* *fulvellum*	Sulfated polysaccharides purified from ethanolic extract by HPLC.	LPS-stimulated RAW 264.7 macrophages and LPS-induced zebrafish embryonic cells.	Suppresses production of NO, TNFα, IL-1β and IL-6, downregulates expression of iNOS and COX-2 in LPS-stimulated RAW 264.7 cells, improves survival rate and reduces cell death, reactive oxygen species (ROS) and NO in LPS-stimulated zebrafish at concentration ranges from 25 to 100 µg/mL.	[33]
*Sargassum* *horneri*	Alginic acid purified from ethanolic extract by HPLC and GC/MS.	LPS-stimulated RAW 264.7 and human haCaT cells and particulate-matter-stimulated inflammation in keratinocytes and fibroblasts	Suppresses PGE2 production via COX-2 inhibition, decreases pro-inflammatory cytokines and abates NF-κB and MAPK pathways in the model system at concentration ranges from 50 to 125 µg/mL.	[34]
*Grifola* *frondosa*	Laminarin purified from water extract by dialysis.	LPS-stimulated RAW 264.7 macrophages.	Inhibits NO and PGE2 production, suppresses pro-inflammatory cytokine (TNF-α and IL-6) secretion and inactivates NF-κB pathway at concentration ranges from 50 to 200 µg/mL.	[35]
*Padina* *boryana*	Fucosterol purified from ethanolic extract by HPLC.	Particulate matter and LPS-stimulated RAW 264.7 macrophages.	Inactivates NF-kB and MPAK pathways and suppresses iNOS, COX-2, pro-inflammatory cytokines and PGE2 mRNA expression at concentration ranges from 12.5 to 50 µg/mL.	[36]
*Stypopodium flabelliforme*	Epitaondiol purified from marine metabolite.	In vitro sPLA2 activity, 12-O-tetradecanoylphorbol-13-acetate (TPA)-induced mouse ear edema model.	Inhibits phospholipase A2 production, suppresses eicosanoid (LTB4 and TXB2) release and reduces TPA-induced mouse ear inflammation at approximately 3.8 µM.	[37]
*Laurencia* *glandulifera*	Neorogioltriol purified from many solvent fractions by HPLC.	LPS-stimulated RAW 264.7 macrophages, DSS-induced colitis in mice.	Suppresses macrophage activation, promotes M2-like anti-inflammatory phenotype and suppresses DSS-induced colitis by reducing tissue damage and pro-inflammatory cytokine production.	[38]
*Dunaliella tertiolecta*	Ergosterol purified from lipid extract by HPLC and analyzed by GC/MS.	LPS- and ConA-stimulated sheep peripheral blood mononuclear cells.	Inhibits pro-inflammatory cytokines (TNF-α, IL-6, IL-1β and IL-10) production at concentration of 0.2 to 0.8 mg/mL.	[39]
*Sargassum* *muticum*	Apo-9′-fucoxanthinone.	LPS-stimulated RAW 264.7 macrophages and LPS-induced zebrafish embryonic cells.	Suppresses mRNA expression of inflammatory mediators such as iNOS, COX-2 and pro inflammatory cytokines, and modulates NF-κB and MPAK signaling pathways	[40]
*Amansia* *multifida*	Lectin purified by sodium salt extraction and HPLC.	Carrageenan-triggered inflammation models in rat.	Reduces parameters of the inflammatory process such as edema formation and leukocyte migration, and modulates levels of proinflammatory cytokines, IL-1β and TNF-α.	[41]
*Bryothamnion* *triquetrum*	Lectin.	Carrageenan-triggered inflammation in rat.	Inhibits vascular and cellular events of an acute inflammatory response, and inhibits neutrophil migration to inflammation sites via suppression of TNF-α and IL-1β production at different concentrations in different models.	[42]
*Ulva* spp.	Peptide fractions purified from enzyme hydrolysate and characterized by FPLC.	LPS and ConA-stimulated rat spleen mononuclear cells.	Modulates TLR4 and the NFκB/p38/JNK pathway at 0.01 g/L to 0.1g/L.	[43]
*Caulerpa peltata*, *Caulerpa racemosa*	Caulerpin purified from ethanolic extract and crystalized from liquid portioning.	DSS-induced colitis in mice.	Reduces inflammatory infiltrates and the levels of the proinflammatory cytokines, increases the levels of the anti-inflammatory cytokine IL-10 and suppresses NF-κB p65 expression.	[44]
*Gracilaria* *opuntia*	Azocinyl morpholinone alkaloid purified from ethanol:methanol extract by HPLC.	Carrageenan-triggered inflammation in rat. In vitro anti-inflammatory model using 5-LOX inhibition assay.	Reduces edema formation by 6 h and exhibits a selective inhibitory effect on COX-2 and 5-LOX activity at a concentration of approximately 0.08 mg/mL.	[45]
*Ulva* *pertusa*	3-Hydroxy-4,7-megastigmadien-9-one purified from aqueous ethanol extract by MPLC.	CpG-stimulated C57BL/6 mice bone-marrow-derived dendritic cells.	Inhibits IL-12 p40, IL-6 and TNF-α production and blocks MAPKs and NF-κB pathways at concentration ranges from 0.1 to 50 µM.	[46]
*Laelia* *undulata*	Floridoside purified from methanolic extract by thin-layer chromatography.	LPS-stimulated BV-2 microglia cells.	Inhibits the production of NO and ROS and downregulates the protein and gene expression levels of iNOS and COX-2 by significantly blocking the phosphorylation of p38 and ERK in LPS-stimulated BV-2 cells at concentration ranges from 10 to 50 µM.	[47]
*Cymopolia* *barbata*	Cymopol and cyclocymopol purified from various non-polar extract through HPLC.	DSS-induced colitis in mice, zebrafish tail wound model and RAW 264.7 macrophages.	Attenuates neutrophil migration and reduces the colon inflammation at the in vitro concentration of 1 to 3 µM and 0.1 to 0.4 g/kg body weight.	[48]
*Sargassum* *siliquastrum*	Sargachromanol G isolated from aqueous methanol and other solvent and then purified from HPLC.	LPS- and RANKL-stimulated RAW 264.7 macrophages	Reduces the expression of pro-inflammatory cytokines, suppresses NO and PGE2 production via inhibition of iNOS and COX-2 and inhibits RANKL-induced activation of NF-κB by suppressing RANKL-mediated IκB-α degradation at concentration ranges from 10 to 40 µM.	[49,50]

**Table 4:** Anti-inflammatory compounds derived from peanut worms.

Marine Source	Species	Bioactive Compounds/Extracts/ Purified	Model	Anti-Inflammatory Activity and Dose	Ref.
Peanut worms	*Sipunculus nudus*	Hot water extract.	Carragenan-induced rat paw oedema model, DSS-induced rat paw oedema model, etc.	Paw edema is reduced by 50–60% within 4h in the test models at concentration of 200 mg/kg body weight.	[120]
	*Phascolosoma* *esculenta*	Oligosaccharide was purified from body wall by enzymatic hydrolysis Sephadex column chromatography. Characterized by mass spec.	Anti-inflammatory mice sepsis model used through intraperitoneal injection of *E. coli*.	Reduces IL1β and TNFα and enhanced anti-oxidant enzyme activity at a dose of 10 to 50 mg/kg body weight	[123]
	*Sipunculus nudus*	Anti-inflammatory peptides were purified from peanut worm powder through enzymatic as well as HPLC and sequenced by Q-TOF-ESI-MS/MS. Same peptides were synthesized in the laboratory.	LPS-induced RAW 264.7 macrophages.	Reduces IL1β and TNFα, and also decreases the expression of iNOS. Decreases the level of NO production at dose ranges from 30 to 120 mM.	[121]
	*Sipunculus nudus*	Collagen peptides were purified from coelomic wall by enzyme hydrolysis and then characterized through SDS and FTIR. Amino acid composition and molecular weight distribution were also determined.	In vitro and in vivo wound healing models were tested.	Enhances wound healing by reducing excessive inflammation in skin of mice through decreasing IL1β and TNFα by using SNCP ointment (2 g/mL).	[122]
